# Effects of High-Fat Diet During Childhood on Precocious Puberty and Gut Microbiota in Mice

**DOI:** 10.3389/fmicb.2022.930747

**Published:** 2022-07-14

**Authors:** Tingbei Bo, Min Liu, Liqiu Tang, Jinzhen Lv, Jing Wen, Dehua Wang

**Affiliations:** ^1^State Key Laboratory of Integrated Management of Pest Insects and Rodents, Institute of Zoology, Chinese Academy of Sciences, Beijing, China; ^2^School of Life Sciences, Shandong University, Qingdao, China; ^3^CAS Center for Excellence in Biotic Interactions, University of Chinese Academy of Sciences, Beijing, China; ^4^College of Life and Environmental Science, Wenzhou University, Wenzhou, China

**Keywords:** precocious puberty, gut microbiota, mice, high-fat diet, mating behavior tendency

## Abstract

Precocious puberty mostly stems from endocrine disorders. However, more and more studies show that a high-fat diet (HFD) is closely related to precocious puberty, but its mechanism is unknown. Since gut microbiota is associated with hormone secretion and obesity, it inspires us to detect the mechanism of gut microbiota in triggering precocious puberty. The model of precocious puberty was established by feeding female mice with an HFD from 21 days old. After puberty, the serum hormone levels, gut microbiome sequencing, and metabolomics were collected. DNA was extracted from feces, and the V3–V4 region of the bacterial 16S rRNA gene was amplified, followed by microbial composition analysis. Subsequently, associations between precocious puberty and the microbiota were determined. We found that (1) HFD after weaning caused precocious puberty, increased serum estradiol, leptin, deoxycholic acid (DCA), and gonadotropin-releasing hormone (GnRH) in the hypothalamus; (2) Through correlation analysis, we found that GnRH was positively correlated with *Desulfovibrio, Lachnoclostridium, GCA-900066575, Streptococcus, Anaerotruncus*, and *Bifidobacterium*, suggesting that these bacteria may have a role in promoting sexual development. (3) “HFD-microbiota” transplantation promoted the precocious puberty of mice. (4) Estrogen changes the composition and proportion of gut microbiota and promotes precocious puberty. Therefore, the effect of HFD on precocious puberty is regulated by the interaction of gut microbiota and hormones.

## Introduction

Puberty is mainly regulated by the central nervous system and is achieved through the hypothalamus-pituitary-gonad (HPG) axis (Kalantaridou, 2002). The enhancement of gonadotropin-releasing hormone (GnRH) neurosecretion promotes the onset of puberty (Chehab, 2014). However, with the improvement of living standards, adolescent obesity, and precocious puberty cases occur from time to time. Precocious puberty can lead to rapid bone development, premature cessation of linear growth, obesity, short stature, increased risk of diabetes, and cancer (Lakshman et al., 2008; Ritte et al., 2013), and some psychological problems, including anxiety, depression, and social disorder. Precocious puberty is usually promoted by obesity, but HFD feeding can also cause early estrus in low weight and body fat mice (Frisch et al., 1977). Therefore, we designed this study to further explore the mechanism of how high-fat diets (HFDs) influence sexual development.

Puberty is regulated by hormones. It was known that in females, increased GnRH secretion can up-regulate the HPG axis, promote luteinizing hormone (LH) and follicle-stimulating hormone (FSH) secretion, and affect the growth and development of sexual organs. At the same time, downstream hormone-estradiol can also promote sexual development and advance the vaginal opening time (Steele and Judith, 1974; Ojeda et al., 1986). The promotion of obesity on sexual development is often related to leptin, which can stimulate the release of luteinizing hormones LH and FSH, and also participate in the early onset of puberty. Previous studies in mice have found that the precocious puberty induced by early overnutrition is mainly attributed to the changes in hypothalamic neural pathways. The increased expression of kisspeptin or its coding gene KISS1 in the hypothalamus promotes the vaginal opening. Studies have shown that KISS1 knockout mice have abnormal puberty, cannot start estrus, have immature reproductive organs, and have low levels of sex steroids and gonadotropins (Funes et al., 2003; Kaiser and Kuohung, 2005; Tassigny et al., 2007).

Gut microbiota plays an important role in host energy metabolism (Tilg and Gasbarrini, 2013; Shanahan et al., 2017). Obesity is often accompanied by gut microbiota disorder, such as the increase in the proportion of Firmicutes/Bacteroides (Ley et al., 2005; Chang et al., 2017). Studies in mice have shown that gut microbiota may affect sex hormones, such as blood testosterone. In turn, sex steroids were able to change the physiological function of gut bacteria (Thackray, 2019). Although the relationship between precocious puberty and gut microbiota has been explored in human studies (Li et al., 2021), the mechanism of the microbiota in regulating precocity is unknown. In this study, we established a precocious puberty model of female mice by feeding high-fat food and analyzed the changes in microbiota and metabolites by 16S rRNA sequencing and metabolomics. Then, fecal microbiota transplantation was used to explore the relationship between HFD, microbiota, hormones, and precocity.

## Materials and Methods

### Animal Experiments and Study Design

All animals were licensed under the Animal Care and Use Committee of the Institute of Zoology, the Chinese Academy of Sciences, China. C57 mice (21 days old) were bought from SPF Biotechnology Co., Ltd. (Beijing, China). The mice were housed individually in a plastic cage and were maintained at room temperature, under a photoperiod of 16L:8D.

Design 1: To study the effect of HFD on sexual development after weaning, 16 female 21 days-old mice were fed a chow diet (fat: 6.2%, carbohydrate: 35.6%, protein: 20.8%, and the calorific value: 17.6 KJ/g), and 16 female 21 days-old mice were fed with HFD (fat: 60%, carbohydrate: 20%, protein: 20%, and the calorific value: 22.0 KJ/g). After attaining the age of 35 days (all mice had achieved puberty by this time), eight mice were randomly sacrificed in the HFD group (*n* = 8, named HFD). Eight mice from the chow food group were also randomly executed (*n* = 8, named CHD). Then, all of the mice were fed a chow food diet until they reached adulthood (*n* = 8, named HF-C and CH-C).

Design 2: To study the effect of gut microbiota on precocious puberty, 10 female 21 days-old mice with chow food, were treated with antibiotics (containing 100 μg/ml neomycin, 50 μg/ml streptomycin, and 100 U/ml penicillin; Sigma, Germany) for 1 week, then transplanted the donors’ microbiota 3 days later. The feces were collected from donors: HFD and CHD mice (design 1), diluted (200 mg/10 ml) in saline, and then, a 50 μl suspension was delivered by intragastric gavage to each bacteria-restricted recipient mouse twice a day (*n* = 5 mice/group, named HFD-FMT and CON-FMT).

Design 3: Twelve female 21 days-old mice were fed chow food, 6 mice were subcutaneously injected with estradiol (5 μg/d, *n* = 6, named EST), and 6 were subcutaneously injected with normal saline (*n* = 6, named CON).

### Determination of Precocious Puberty

The female mice were held in place and their tails were lifted to expose the vulva, then the opening of the vulva was observed. When the symptoms of estrus (vaginal opening, moist, mucus secretion, mucous membrane crinkle wall swelling) appeared, we determined that the mouse was in puberty (Hoffmann, 2018).

### Measurements of Proteins Related to Reproduction by Western Blotting

Hypothalamus was homogenized in Radio Immunoprecipitation Assay Lysis buffer (RIPA) buffer and cleared by centrifugation, according to the standard techniques. Western blots of whole tissue lysates were probed with primary antibodies against Kisspeptin (ab226786, Abcam, Cambridge, United Kingdom), GnRH (PA5-97047, Thermo Fisher Scientific, Waltham, MA, United States), β-Tubulin (A01030, Abbkine, CA, United States), GAPDH (A01020; Abbkine, CA, United States). The secondary antibody used was either peroxidase-conjugated goat anti-rabbit IgG (111-035-003; Jackson, West Grove, PA, United States), or peroxidase-conjugated goat anti-mice IgG (115-035-003; Jackson, West Grove, PA, United States). Protein markers (20351ES76; Shanghai Yisheng, China) were added on both sides of each gel to verify bands. The polyvinylidene fluoride (PVDF) membranes were detected by enhanced chemoluminescence (Beyotime, China). The bands were analyzed using Image LabTM Software (Bio-Rab Laboratories, Hercules, CA, United States), were normalized to β-Tubulin or GAPDH, and were expressed as relative units (RU).

### Total RNA Extraction and Real-Time Polymerase Chain Reaction of FshR and LhR Genes

To determine the expression level of receptors of FSH and LH (FshR and LhR), we took unilateral ovaries after the animals were killed. Total RNA was extracted from liquid nitrogen-frozen ovaries using TRIzol Regent (Takara Bio, Inc., Shiga, Japan). RNA integrity and quality were determined by agarose gel electrophoresis (1%) and spectrometry (A260/A280), respectively. A commercial reverse transcription (RT) kit (Takara Bio, Inc.) was used for the synthesis of cDNA. The RT products (cDNA) were stored at –20°C for relative quantification by PCR. For real-time quantitative polymerase chain reaction (RT-qPCR), every reaction was performed in triplicate using SYBR green kits on an Applied Biosystems ABI 7500 system (Thermo Fisher Scientific, Waltham, MA, United States). The expression levels of all genes were normalized to that of GAPDH using the 2^–ΔΔCT^ method. The sequences of primers used for RT-qPCR are listed in Supplementary Table 1.

### Measurements of Estradiol, Leptin, and Deoxycholic Acid

Serum estradiol concentrations were quantified using a 17 beta Estradiol ELISA kit (ab 108667, Abcam, Cambridge, United Kingdom) according to the instructions. The minimum detected concentration of the kit was 8.68 pg/ml.

Serum leptin concentrations were quantified using a Leptin mouse ELISA kit (ab 100718, Abcam, Cambridge, United Kingdom) according to the given instructions. The minimum detected concentration of the kit was 4 pg/ml for leptin.

Serum DCA concentrations were quantified using an ELISA kit (CESO89Ge, Cloud-clone, China) according to the given instructions. The minimum detected concentration of the kit was 4.99 ng/ml for DCA.

### Microbiota DNA Extraction, Evaluation, and Amplification

After sacrificing mice, we got their cecal contents. DNA from cecal contents was extracted by using cetyltrimethylammonium bromide (CTAB), phenol-chloroform mixture (phenol:chloroform:isoamyl alcohol = 25:24:1). DNA purity was also assessed by absorbance on a NanoDrop 2000 (Thermo Fisher Scientific, Carlsbad, CA, United States) by measuring the A260/A280 ratio. Our target was the V3–V4 hyper-variable region of the bacterial 16S rRNA gene. PCR was started immediately after the DNA was extracted. The 16S rRNA V3–V4 amplicon was amplified by using 2 × Taq PCR MasterMix (Tiangen, Beijing, China). Two universal bacterial 16S rRNA gene amplicon PCR primers (PAGE purified) were used: forward primer-341F (CCTAYGGGRBGCASCAG) and reverse primer-806R (GGACTACNNGGGTATCTAAT). PCR products were pooled and purified using Agencourt AMPure XP magnetic beads (Beckman) according to the manufacturer’s instructions. TruSeq^®^ DNA PCR-Free Sample Preparation Kit was used to construct the library. The constructed library was quantified by Qubit and Q-PCR. Finally, the library was sequenced on an Illumina NovaSeq platform and 250 bp paired-end reads were generated.

### 16S rRNA Gene Amplicon Sequencing Analysis

Paired-end reads were merged using FLASH (Version 1.2.11).^1^ Quality filtering on the raw tags was performed using the fastp (Version 0.20.0) software to obtain high-quality Clean Tags. The Clean Tags were compared with the reference database (Silva database for 16S)^2^ using Vsearch (Version 2.15.0) to detect the chimera sequences, and then the chimera sequences were removed to obtain the Effective Tags. For the Effective Tags obtained previously, denoise was performed with DADA2 or deblur module in the QIIME2 software (Version QIIME2-202006) to obtain initial Amplicon Sequence Variants (ASVs) (default: DADA2), and then ASVs with abundance < 5 were filtered out. Species annotation was performed using QIIME2 software. In order to study the phylogenetic relationship of each ASV and the differences of the dominant species among different samples (groups), multiple sequence alignment was performed using QIIME2 software. The absolute abundance of ASVs was normalized using a standard sequence number corresponding to the sample with the least sequences. Subsequent analyses of alpha diversity and beta diversity were all performed based on the output normalized data. In order to analyze the diversity, richness, and uniformity of the communities in the sample, alpha diversity was calculated in QIIME2, including the Shannon index. Principal Coordinate Analysis (PCoA) was performed to obtain principal coordinates and visualize differences of samples in complex multi-dimensional data. To study the significance of the differences in community structure between groups, the adonis and anosim functions in the QIIME2 software were used to do the analysis. To find out the significantly different species at each taxonomic level (phylum, class, order, family, genus, species), the R software (Version 3.5.3) was used to do MetaStat and *T*-test analysis. The linear discriminant analysis effect size (LEfSe) software (Version 1.0) was used to do LEfSe analysis [linear discriminant analysis (LDA) score threshold: 4] so as to find out the biomarkers.

### Untargeted Metabolomic Study Based on Liquid Chromatography Tandem Mass Spectrometry

Cecum content (50 mg) was individually ground with liquid nitrogen and the homogenate was resuspended in pre-chilled 80% methanol (−20°C) and then vortexed. The samples were incubated at −20°C for 60 min and then centrifuged at 14,000 *g* and 4°C for 20 min. The supernatants were subsequently transferred to a fresh tube and spun in a vacuum concentrator until dry. The dried metabolite pellets were reconstituted in 60% methanol and analyzed by LC-MS/MS. LC-MS/MS analyses were performed using a Vanquish ultra-high-performance liquid chromatography (UHPLC) system (Thermo Fisher Scientific, Waltham, MA, United States) coupled with an Orbitrap Q Exactive HF-X mass spectrometer (Thermo Fisher Scientific, Waltham, MA, United States) operating in the data-dependent acquisition mode. The raw data files generated by UHPLC-MS/MS were processed using the Compound Discoverer 3.0 (Thermo Fisher Scientific, Waltham, MA, United States) to perform peak alignment, peak picking, and quantitation of each metabolite. Afterward, peak intensities were normalized to the total spectral intensity. The normalized data were used to predict the molecular formula based on additive ions, molecular ion peaks, and fragment ions. Then, the peaks were matched with the mzCloud^3^ and ChemSpider^4^ databases to obtain accurate qualitative and relative quantitative results. The online Kyoto Encyclopedia of Genes and Genomes (KEGG) database was used to identify metabolites by matching the molecular mass data. Finally, metabolites for separating the models were selected with the following requirements: variable importance in projection (VIP) > 1 and | P(corr)| ≥ 0.5 with 95% jack-knifed confidence intervals. The Student’s *t*-test was applied to further analyze the intergroup significance of the selected metabolites. Pathway analysis and enrichment analysis of differential metabolites were conducted on the MetaboAnalyst web server.^5^

### Statistical Analysis

All statistical analyses and figures were performed using IBM SPSS 22.0. (IBM Corporation, United States) and GraphPad Prism version 9.00 (GraphPad Software, San Diego, CA, United States). Data were used for the repeated-measures ANOVA, independent *t*-test, and Kruskal–Wallis test. An independent *t*-test was used to analyze the relative abundance of the phylum. For different abundance analyses in STAMP, Welch’s *t*-test was used for the comparison of the two groups. The correlations between the cecum microbial composition and metabolites and host precocious puberty indexes were assessed by Spearman’s correlation analysis. A probability (*p*) value of < 0.05 was considered statistically significant (*p < 0.05, **p < 0.01, ***p < 0.001).

## Results

### HFD After Weaning Changes the Gut Microbiome and Promotes Precocious Puberty

**FIGURE 1:**
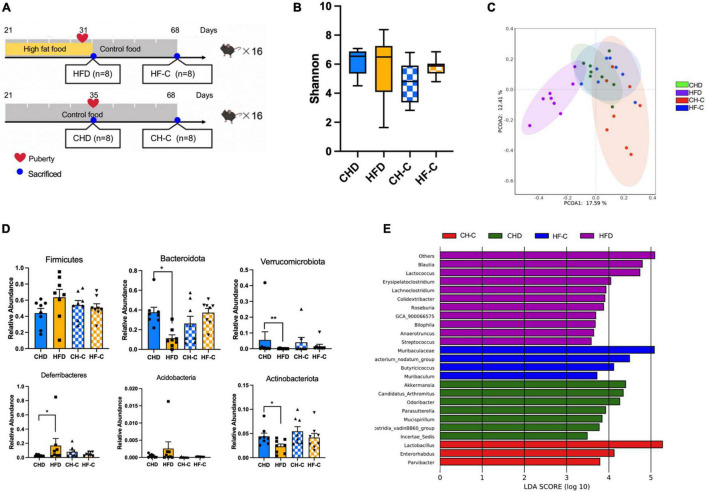
High-fat diet after weaning changes gut microbiota in female mice. **(A)** Experiment design of the study in female mice. **(B)** Shannon diversity of gut microbiota. **(C)** PCoA plot of bray_curtis distance of gut microbiota. **(D)** The difference of the abundance at phylum level. **(E)** Results from LEfSe analysis, showing the most differentially abundant genus enriched in 4 groups. Data are means ± SEM. *P < 0.05, **P < 0.01, and ***P < 0.001 (*n* = 8).

To investigate whether HFD during childhood causes changes in the gut microbiota, we collected the cecal content of four groups of mice (Figure 1A). There was no difference in the Shannon index (alpha diversity) among the four groups (Figure 1B). Profiling of the microbiota composition, followed by PCoA based on Bray–Curtis distance, showed major alterations in the microbiota of HFD animals (Adonis, *p* < 0.05, Figure 1C). Firmicutes and Bacteroidetes were the most abundant phylum in all samples (on average 47.80 and 29.70%) (Supplementary Figure 1A). At the phylum level, compared with CHD mice, Bacteroidetes, Verrucomicrobia, and Actinobacteria were decreased in HFD mice, while Deferribacteres was increased in the HFD mice (Figure 1D). At the genus level, *Bilophia*, *Lactococcuus, Lachnoclostridium, Streptococcus, GCA_900066575*, and *Anaerotruncus* were enriched in the HFD group; *Akkermansia* and *Lactobacillus* were enriched in the CHD and CH-C groups (Figure 1E).

**FIGURE 2:**
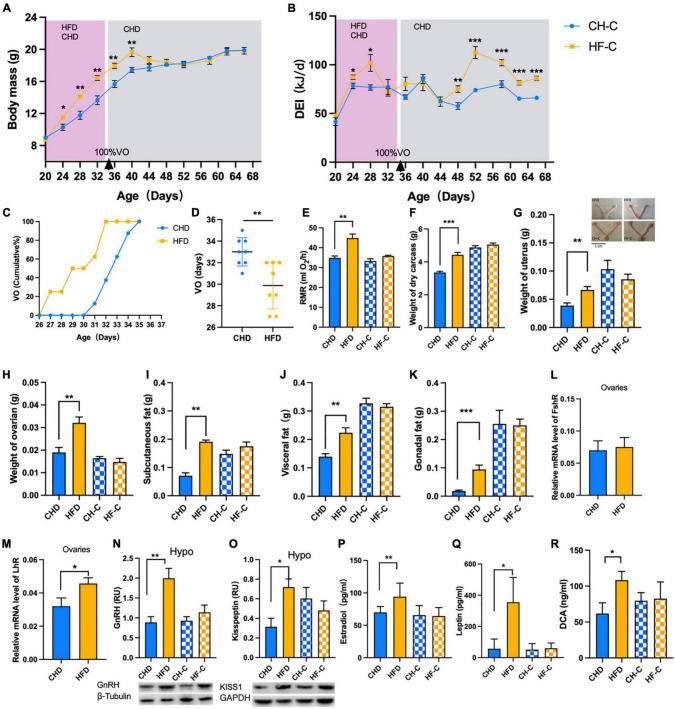
High-fat diet after weaning promotes precocious puberty in female mice. **(A)** Body mass of the mice in CH-C and HF-C groups (*n* = 8). **(B)** Daily energy intake of the mice in CH-C and HF-C groups (*n* = 8). **(C)** Cumulative percentage of vaginal opening (VO) of the mice (*n* = 16). **(D)** Vaginal opening days of the mice (*n* = 16). **(E)** The resting metabolic rate (RMR) of the mice (*n* = 8). **(F)** Weight of dry carcass of the mice (*n* = 8). **(G,H)** Weight of ovaries and uterus of the mice (*n* = 8). **(I–K)** The weight of subcutaneous fat, visceral fat and gonadal fat of the mice(*n* = 8). **(L,M)** Relative mRNA level of FshR and LhR (*n* = 8). **(N,O)** Content of GnRH and Kisspeptin1 in hypothalamus (*n* = 8). **(P–R)** Concentration of estradiol, leptin and DCA concentration in serum (*n* = 8). Data are means ± SEM. *P < 0.05, **P < 0.01, and ***P < 0.001.

**FIGURE 3:**
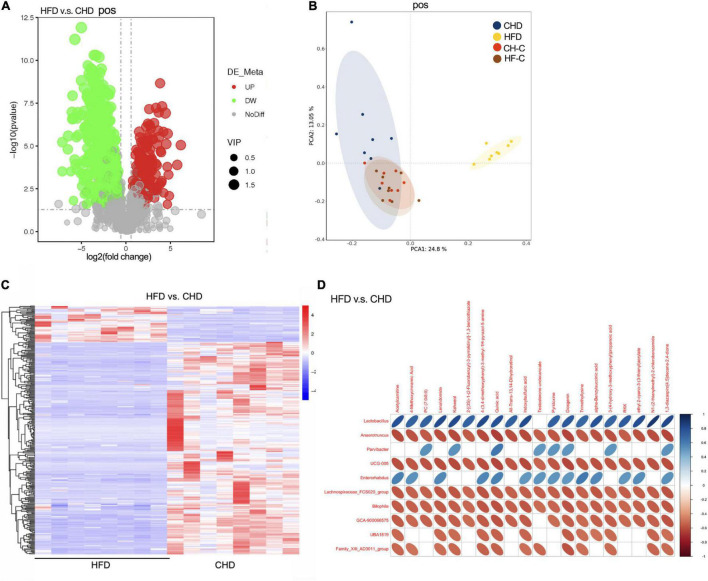
High-fat diet after weaning changes metabolic communities of the mice. **(A)** Score plot of LC-MS (postive) data with 1176 metabolite signals detected. **(B)** PCA score plot of metabolomic data for 4 groups obtained by LC-MS (postive). **(C)** Heat plot of metabolomic data in HFD and CHD groups (postive). **(D)** Correlation analysis between cecum metabolites and microbiota in HFD and CHD groups (postive). Data are means ± SEM. *P < 0.05, **P < 0.01, and ***P < 0.001 (*n* = 8).

The mice were in the period of growth therefore weight of both groups increased significantly over time (Figure 2A), and the body weight of HF-C mice was higher than that of the CH-C group. For daily energy intake, repeated measures showed significant time effect and treatment effect (Figure 2B). The RMR was higher in HFD mice than CHD mice (Figure 2E). Due to the obesity caused by high-fat food, the carcass dry weight, subcutaneous fat, visceral fat, and gonadal fat of the HFD group were significantly higher than those of the CHD group (Figures 2F–K). To identify precocious puberty, we measured the time of vaginal opening and found that for HFD mice, it was significantly earlier than for CHD mice (Figures 2C,D). The weight of ovaries and uterus (Figures 2G,H) and LhR expression of ovaries (Figure 2M) in the HFD group were significantly higher than in the CHD group while there was no difference in FshR (Figure 3L). The HFD also increased the expression of GnRH and Kisspeptin in the hypothalamus (Figures 2N,O). We measured the serum estradiol and leptin. The content of estradiol and leptin in the HFD group was significantly higher than that in the CHD group (Figures 2P,Q). Finally, we measured DCA in serum, which is the secondary bile acids produced by microbes. The DCA concentration in the HFD group was significantly higher than in the CHD group (Figure 2R).

### HFD After Weaning Changes Metabolic

To further study the effect of HFD on the mice, the cecal content metabolic profiles of the four groups were acquired by LC-MS. LC-MS/MS (negative and positive) detected a total of 663 and 1,176 biomarker metabolites, respectively. Compared with CHD, it was found that 146 biomarker metabolites were up and 361 biomarker metabolites were down in HFD mice (positive Figure 3A; negative, 96 up and 344 down, Supplementary Figure 1B). As shown by the PCA score-plots presented in Figure 3B (or Supplementary Figure 1C), which distinguished metabolic communities based on cecum sampling, the metabolic communities were clustered. HFD group was significantly separated from the other three groups. Further, the heat map of differential metabolites between the HFD and CHD groups showed a significant difference (Figure 3C, positive and Supplementary Figure 1D, negative).

Metabolomics has been shown as an important tool to reveal the potential crosstalk of host and gut microbiota. Therefore, correlations between the top 20 metabolites and genus-level microbiota with significant differences in HFD and CHD mice were obtained *via* Spearman correlation analysis (Figure 3D, positive and Supplementary Figure 1E, negative). We found that pyridoxine was negatively correlated with *Anaerotruncus*, *Bilophila*, and GCA-900066575, which were enriched in the HFD group. Through the results of metabonomics, we found that the content of pyridoxine in CHD was much higher than that of the HFD group (Supplementary Figure 1F). Spearman correlation showed that pyridoxine had a negative correlation to body weight, uterine weight, and Kisspeptin expression (Supplementary Figure 1G). This suggested that pyridoxine is negatively correlated with precocious puberty.

### Gut Microbiota Is the Key Factor to Promote Precocious Puberty

**FIGURE 4:**
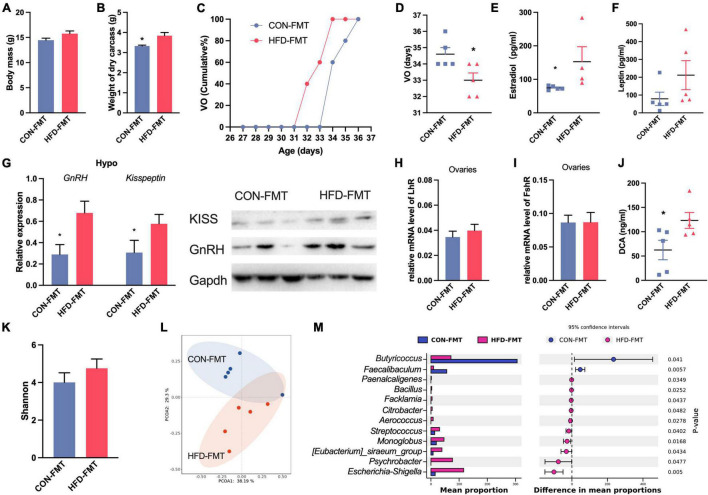
Fecal microbiota affect precocious puberty and hormone. **(A)** Body mass. **(B)** Weight of dry carcass. **(C)** Cumulative percentage of vaginal opening (VO). **(D)** Vaginal opening days. **(E,F)** Concentration of Estradiol and leptin in serum. **(G)** Expression of GnRH and Kisspeptin1 in hypothalamus. **(H,I)** Relative mRNA level of FshR and LhR in ovaries. **(J)** DCA concentration in serum. **(K)** Shannon diversity of gut microbiota. **(L)** PCoA plot of bray_curtis distance of gut microbiota. **(M)** Difference of abundance of genus in gut microbiota (STAMP). Data are means ± SEM. *P < 0.05, **P < 0.01, and ***P < 0.001 (*n* = 5).

We chose the CHD group and HFD group in design 1 as the gut microbiota donor mice. After fecal microbiota transplantation, the weight of the dry carcass was increased in HFD-FMT mice (Figures 4A,B), and the vaginal opening time of HFD-FMT mice was advanced (Figures 4C,D). In the HFT-FMT group, the serum estradiol was higher than in the CON-FMT mice (Figure 4E), while leptin was not affected (Figure 4F). Expression of GnRH and Kisspeptin in the hypothalamus of HFT-FMT were significantly higher than in the CON-FMT group (Figure 4G). The expression of LhR in the ovaries of the HFD-FMT group was significantly higher than in the CON-FMT group (Figures 4H,I). The DCA in the HFD-FMT group was higher than in the CON-FMT group (Figure 4J). The alpha diversity of the two groups was no different (Figure 4K). PCoA based on Bray–Curtis distance showed major alterations in the microbiota between groups (Figure 4L). At the genus level, *Bacillus, Streptococcus, Aerococcus, Facklamia, Psychrobacter*, and *Monoglobus* were enriched in the HFD-FMT group, while *Faecalibaculum* and *Butyricicoccus* were enriched in the CON-FMT group (Figure 4M).

### Effects of Estrogen on Gut Microbiota

**FIGURE 5:**
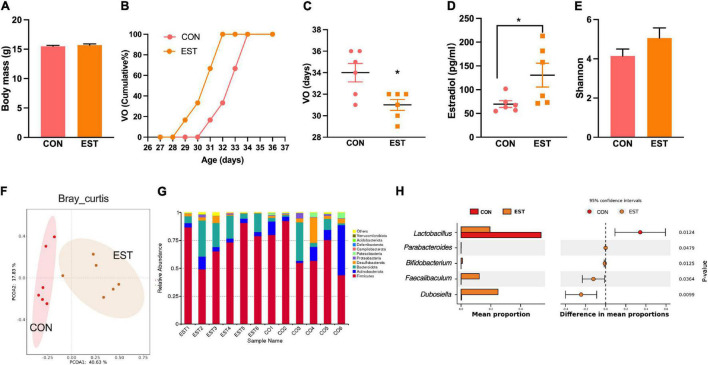
Estradiol affects precocious puberty and gut microbiota. **(A)** Bodymass of mice. **(B)** Cumulative percentage of vaginal opening (VO). **(C)** Vaginal opening days. **(D)** Concentration of Estradiol in serum. **(E)** Shannon diversity of gut microbiota in mice. **(F)** PCoA plot of bray_curtis distance of gut microbiota in mice. **(G)** Bar plot of relative abundant phylum of gut microbiota in mice. **(H)** Results from STAMP analysis, showing the most differentially abundant taxa between the two groups. Data are means ± SEM. *P < 0.05, **P < 0.01, and ***P < 0.001 (*n* = 6).

To test whether the estrogen associated with precocious puberty also affects the gut microbiota, we designed estradiol treatment to assess gut microbiota changes. The body mass between the two groups was no different (Figure 5A). The vaginal opening time of EST mice was advanced (Figures 5B,C). Estradiol in the EST group was significantly higher than that in the CON group (Figure 5D). There was no significant difference in alpha diversity between the two groups (Figure 5E). PCoA with Bray–Curtis distance revealed separation of the two groups and that there was a significant difference between EST and CON (Adonis, *p* < 0.05, Figure 5F). Estradiol significantly changed the composition and structure of gut microbiota (Figure 5G). We found that *Dubosiella* and *Faecalibaculum* were enriched in the EST treatment group (Figure 5H), while *Lactobacillus* was enriched in the CON group.

**FIGURE 6:**
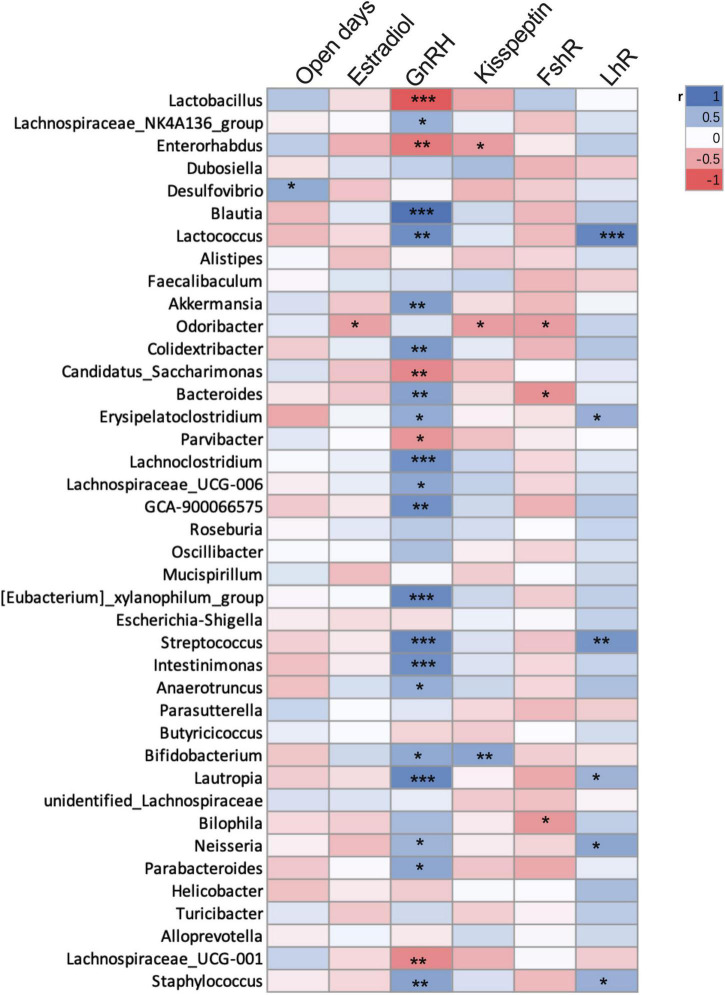
Correlation between precocious puberty phenotype and bacterial abundance of mice in the 3 experiments. Different color blocks represent Spearman correlation coefficients, blue indicates positive correlation, and red indicates negative correlation. *P < 0.05; **P < 0.01. (mice were from CHD, HFD, CON-FMT, HFD-FMT, EST and CON groups, *n* = 38).

Based on the above three experiments, we analyzed the correlation between precocious puberty phenotype and genus level bacterial abundance of mice (Spearman, *n* = 38, Figure 6). We found that GnRH was positively correlated with *Blautia, Lactococcus, Akkermansia, Colidextribacter, Lachnoclostridium, GCA-900066575, Streptococcus, Bacteroides, Intestinimonas, Anaerotruncus*, and *Bifidobacterium;* while *Lactobacillus, Enterorhabdus*, and *Lachnospiraceae* were negatively correlated with GnRH (*p* < 0.05).

## Discussion

### HFD Promotes Precocious Puberty and Changes Gut Microbiota

Consistent with previous studies (Heras et al., 2020), we found that HFD mice started puberty earlier. Ullah et al. (2019) found that HFD feeding to dams during lactation and to pups post-weaning triggers body weight gain, body fat deposition, and induces precocious puberty. Unlike other studies, we did not look into HFD during pregnancy and lactation but instead focused on “childhood” after weaning. This is a critical period for the growth and development of mice, especially for shaping the gut microbiota. The established idea is that precocious puberty is promoted by obesity, but studies have shown that HFD feeding can cause early estrus in low weight and body fat mice (Frisch et al., 1977). It seems that HFD feeding may affect some hypothalamic neurocircuitry or gut microbiota involved in metabolism and reproduction. Here, we found that HFD increased the GnRH level which led to changes in the gut microbiota and the development of precocious puberty.

In our results, HFD increased the abundance of *Desulfovibrio, Lachnoclostridium, Bilophia*, and *Anaerotruncus*. The abundance of *Desulfovibrio* is high in obese mice, which always induces intestinal inflammation (Petersen et al., 2019; Zhang et al., 2020), which may indirectly promote precocious puberty by promoting obesity. Additionally, *Anaerotruncus* is harmful to intestinal health and can easily cause obesity (Xu et al., 2017), but its effect on sexual development has not been reported. Correlation analysis showed that GnRH was positively correlated with *Bacteroides*, *Blautia, Akkermansia, Lactococcus, Colidextribacter, Lachnoclostridium, GCA-900066575, Streptococcus, Bacteroides, Intestinimonas, Anaerotruncus*, and *Bifidobacterium*, suggesting that these bacteria may have a role in promoting sexual development. However, many studies have shown that *Akkermansia*, *Bacteroides, Oscillibacter*, *Intestinimonas*, and *Bifidobacterium* are negatively associated with obesity (Thingholm et al., 2019; Depommier et al., 2020; Sroka-Oleksiak et al., 2020). It suggests that “obesity microbiota” and “precocious puberty microbiota” were not always consistent, so the regulation of HFD-induced precocious puberty by bacteria is not always related to obesity. Additionally, pyridoxine was related negatively to body weight, uterine weight, and Kisspeptin expression, which suggested that pyridoxine is negatively correlated with precocious puberty. Pyridoxine is involved in the metabolism of vitamin B6 (K00868, KEGG), which is related to the metabolism of estrogen in women (Luhby et al., 1973). Therefore, the precocious puberty of HFD mice may be mediated by increasing *Bilophia, GCA-900066575*, and *Anaerotruncus* and decreasing pyridoxine.

### Gut Microbiota Transplantation Promotes Precocious Puberty

Studies have found that gut microbiota was related to precocious puberty. For example, in the central precocious puberty (CPP) girls, *Ruminococcus bromii, Ruminococcus gnavus*, and *Clostridium leptum* were enriched (Khoo and Perera, 2005). In cases of CPP, *Akkermansia* is negatively correlated with FSH and LH levels (Li et al., 2021). In our results, *Aerococcus, Streptococcus*, and *Bacillus* were enriched after “HFD-microbiota” was transplanted. These bacteria affect the steady-state of gut microbiota, leading to inflammation. Through FMT, we found that HFD-microbiota promoted the vaginal opening while the mechanism of gut microbiota on precocious puberty is unknown, which may be through direct or indirect ways. Studies showed the gut microbiota modulates local and systemic levels of sex steroids by producing special enzymes, for example, the microbiome can modify host-derived molecules such as bile and sex steroids (Ridlon and Bajaj, 2015; Oliphant and Allen-Vercoe, 2019; Ridlon, 2020). In our study, we also found that the concentration of secondary bile acids and estradiol increased in the HFD-microbiota transplanted mice. Additionally, “HFD-microbiota” increased the leptin and the expression of HPG axis-related genes (GnRH and kisspeptin). Studies have shown that microbiota metabolites (mainly butyric acid and propionic acid) are related to leptin gene expression and participate in the regulation of puberty (Lin et al., 2012). Therefore, we suggest that through secreting neurotransmission-related metabolites, such as butyric acid and propionic acid, the bacteria could trigger the earlier onset of puberty by activating HPGA.

### Interaction Between Hormones and Gut Microbiota Promotes Precocious Puberty

The change of hormones is one of the reasons that cause obesity and promote precocious puberty. Our results also confirmed the view that estrogen injection led to the early vaginal opening in mice, which did not increase body weight. Although estrogen can directly act on reproductive organs and promote puberty, its effect on the intestinal tract cannot be ignored. The intestinal epithelium expresses sex steroid receptors, such as the G-protein-coupled estrogen receptor GPER-1 and nuclear estrogen receptors α (ERα) and β (ERβ). Estrogen increases the expression of Toll-like receptors (TLRs) and proinflammatory cytokines but also suppresses inflammation through ERβ (Pace and Watnick, 2021). In women, increased estradiol was correlated with an increase in Bacteroidetes and a decrease in Firmicutes. Our results showed that *Dubosiella, Faecalibaculum*, and *Bifidobacterium* were increased significantly in the EST group, which were also found in the central precocious puberty girls (Li et al., 2021). *Bifidobacterium* can reduce the level of inflammation (Sun et al., 2020), which was consistent with the anti-inflammatory effect of estrogen. One study found that *Faecalibaculum* and *Lachnospiraceae* were significantly correlated with proteins implicated in mitochondrial energy metabolism (D’Amato et al., 2020). Therefore, estrogen can affect gut microbiota and energy metabolism.

The interaction between hormones and gut microbiota helps in maintaining physiological stability. Gut microbiota regulates sex hormone levels by producing enzymes and releases active estrogen by releasing β-glucuronidase to dissociate the estrogen-bile acid complex (Rizzetto et al., 2018; Pace and Watnick, 2021). In human studies, through the determination of hormones in patients with central precocious puberty, it was found that *Parabacteroides* were positively correlated with LH-releasing hormone (Li et al., 2021). In our gut microbiota transplantation experiment, we found that “HFD-microbiota” can significantly increase the content of estradiol and expression of GnRH and KISS1 in the hypothalamus. Through large sample correlation analysis, we found that *Dubosiella, Faecalibaculum, Alistipes, GCA-900066575, Lachnoclostridium, Anaerotruncus*, and *Bifidobacterium* were positively correlated with GnRH, suggesting that they could promote puberty. Therefore, the interaction between hormones and gut microbiota may be the mechanism of HFD- induced precocious puberty.

## Conclusion

Precocious puberty is regulated by the interaction of gut microbiota and hormones. HFD in adolescence will affect the balance of gut microbiota and estrogen and induce precocious puberty and promote mating behavior. Our results have a potential significance for the mechanism of precocious puberty in children. Our study also found that “obesity microbiota” and “precocious puberty microbiota” are not always consistent. Therefore, in the future, the prevention of precocious puberty in children who are not obese but prefer HFD cannot be ignored.

## Data Availability Statement

The datasets presented in this study can be found in online repositories. The names of the repository/repositories and accession number(s) can be found in the article/Supplementary Material.

## Ethics Statement

The animal study was reviewed and approved by the Institutional Animal Care and Use Committee of Institute of Zoology (Chinese Academy of Sciences), IOZ-IACUC-2020-090.

## Author Contributions

TB and DW designed the research. TB and ML performed animal experiments and molecular experiments. TB, LT, and JL analyzed the data. TB, JW, and DW wrote the manuscript. All authors contributed to the article and approved the submitted version.

## Conflict of Interest

The authors declare that the research was conducted in the absence of any commercial or financial relationships that could be construed as a potential conflict of interest.

## Publisher’s Note

All claims expressed in this article are solely those of the authors and do not necessarily represent those of their affiliated organizations, or those of the publisher, the editors and the reviewers. Any product that may be evaluated in this article, or claim that may be made by its manufacturer, is not guaranteed or endorsed by the publisher.

## Footnotes

1
http://ccb.jhu.edu/software/FLASH/

2
https://www.arb-silva.de/

3
https://www.mzcloud.org/

4
http://www.chemspider.com/

5
http://www.metaboanalyst.ca/faces/home.xhtml
