# Supplementary material for: Effects of High-Fat Diet During Childhood on Precocious Puberty and Gut Microbiota in Mice
Source: Front Microbiol. 2022 Jul 14;13:930747. doi: 10.3389/fmicb.2022.930747 (PMC9329965; doi:10.3389/fmicb.2022.930747)
Supplement: Supplementary file 1 [file Table_1.docx]

**Supplementary**

**Tables**

**Table S1. The primer sequence of different genes in RT-PCR.**

| **Genes** | **Primer sequence (5'-3')** | **Product(bp)** | **GenBank accession** |
| --- | --- | --- | --- |
| FshR | F:CCCATCTTTGGCATCAGTAGC | 208 | NM_013523.3 |
|  | R:GCTTGGCAATCTTGGTGTCTC |  |  |
| LhR | F:ACAGGAGAACACAACCAAAC | 203 | NM_001364898.1 |
|  | R:GAAGGAGACAGCAAACTATGAC |  |  |
| GAPDH | F:CATCAACGGGAAGCCCATC | 211 | NM_008084.2 |
|  | R:CTCGTGGTTCACACCCATC |  |  |

**Table S2. The different indexes of alpha diversity in CHD, HFD, CH-C and HF-C groups.**

| **group** |  | **observed_species** | **shannon** | **simpson** | **chao1** | **PD_whole_tree** |
| --- | --- | --- | --- | --- | --- | --- |
| **CHD** | **Mean** | 785.88 | 5.92 | 0.94 | 918.97 | 89.22 |
|  | **SEM** | 70.49 | 0.30 | 0.02 | 74.08 | 5.48 |
| **HFD** | **Mean** | 794.38 | 5.44 | 0.83 | 904.84 | 85.34 |
|  | **SEM** | 156.26 | 0.77 | 0.09 | 160.27 | 15.61 |
|  | **p vaule** | 1 | 1 | 0.721 | 1 | 0.645 |
|  |  |  |  |  |  |  |
| **CH-C** | **Mean** | 483.63 | 4.47 | 0.82 | 589.11 | 58.79 |
|  | **SEM** | 33.60 | 0.44 | 0.05 | 42.88 | 4.94 |
| **HF-C** | **Mean** | 611.11 | 5.59 | 0.93 | 731.31 | 72.79 |
|  | **SEM** | 42.62 | 0.19 | 0.01 | 51.67 | 8.28 |
|  | **p vaule** | 0.015 | 0.027 | 0.2 | 0.059 | 0.2 |

Independent-sample Mann-Whitney test.


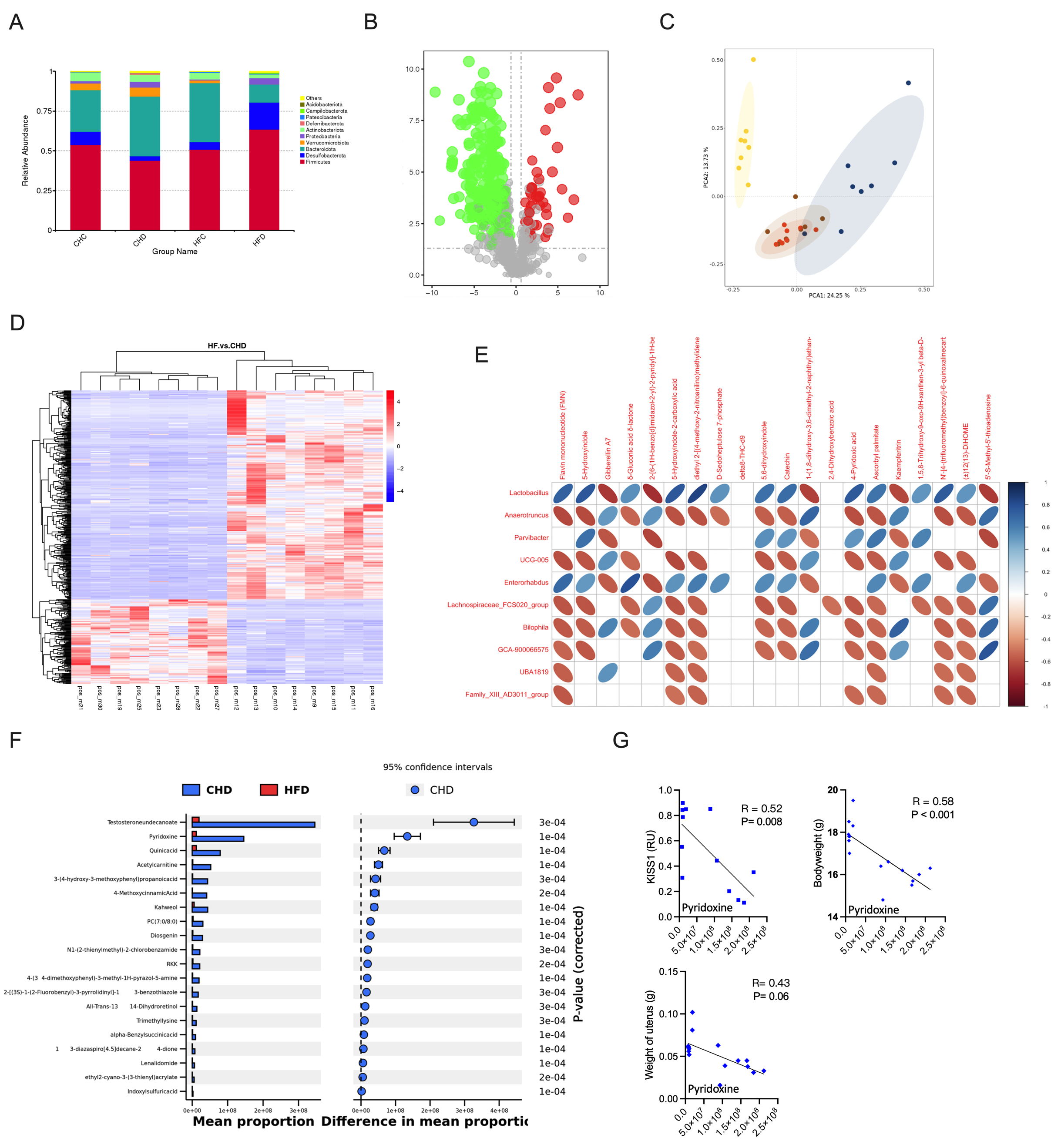


**Figure S1. Supplementary results of gut microbiota and metabolome in Experiment 1**

(A) The difference of the abundance at phylum level in 4 groups. (B) Score plot of LC-MS (negative) data with 1176 metabolite signals detected. (C) PCA score plot of metabolomic data for 4 groups obtained by LC-MS (negative). (D) Heat plot of metabolomic data in HFD and CHD groups (negative). (E) Correlation analysis between cecum metabolites (Negative) and microbiota in HFD and CHD groups. (F) Difference of abundance of metabolites in CHD and HFD groups (STAMP). (G) Correlation analysis between Pyridoxine and precocious puberty indexes. p < 0.05 indicates significant correlation. Data are means ± SEM. *p<0.05, **p< 0.01, and ***p<0.001.
